# Notes on the Effects of Poke-Berries on Birds

**Published:** 1895-02

**Authors:** W. E. Rotzell


### NOTES ON THE EFFECTS OF POKE-BERRIES ON BIRDS.

W. E. Rotzell, M. D.

The use of the juice of the poke-berry (*phytolacca decandra*) as a fat-reducing agent is at present quite extensive, but the evidence that these berries reduce body weight without putting the subject on an anti-fat diet is, I think, far from conclusive.

In *The Homœopathic Recorder* for March, 1892, Dr. E. M. Hale claims priority to the discovery of the anti-fat properties of *phytolacca decandra*. He stated that as far back as 1858 he mentioned in the *New Remedies* that birds which fed on these berries become emaciated.

Of the effect of poke-berries upon birds is to what I desire to call attention. Having been always interested in the study of birds, I have watched for several seasons and noted as carefully as possible the effects, as I observed them, of poke-berries (*phytolacca*) upon birds. I have dissected quite a number of birds which have been feeding upon these berries, and have always found them to not only be in the best physical condition, but invariably fat when they had been feeding on the berries to any extent. The species of birds I know to feed on the berries of *phytolacca decandra* are the following: American robin (*Merula migratoria*), wood thrush (*Turdus mustelinus*), brown thrasher (*Harporhynchus rufus*), cat-bird (*Galeoscoptes Carolinensis*), flicker (*Colaptes auratus*), red-headed woodpecker (*Melanerpes erythrocephalus*), cedar bird (*Ampelis cedrorum*), Carolina chickadee (*Parus Carolinensis*), myrtle warbler (*Dendroica coronata*).

Desiring to know the opinion generally held by naturalists on this subject, I wrote to several prominent ornithologists, whose replies I here present:

Dr. R. W. Shufeldt, United States Army, of the Smithsonian Institute, Washington, a recognized authority on birds, and whose observations cannot be questioned, writes: "I have shot a variety of species of birds that have been feeding on the ripe berries of *phytolacca decandra*. * * * Very often I have shot both robins and flickers that have been absolutely gorged with the dead-ripe fruit of this plant. Such specimens I have not only eaten, but skinned, dissected, and otherwise examined. From the gizzard to the vent the contents of the entire intestinal tract have been stained with the deep claret-colored or carmine-colored juice of the berries, as is the circlet of feathers surrounding the anal aperture. These birds at such times are not always well nourished, but often fat and otherwise in excellent condition."

Prof. H. Justin Roddy, of the Millersville State Normal School, well known as an ornithologist, and who has given especial attention to the subject of bird-foods, wrote me as follows:

"I can say that in the large series of robins, cat-birds, thrushes, and other poke-berry (*phytolacca*) eating birds I have taken I have invariably found them very fat. So fat sometimes that they could not be used as specimens."

Dr. A. K. Fisher, of the Department of Agriculture, wrote me that he did not ever remember having seen a bird in an emaciated condition that had been feeding on *phytolacca decandra* berries.

I also consulted Mr. Witmer Stone, curator of birds at the Academy of Natural Sciences, Philadelphia, and his experience on this subject corresponds practically to those of the above. All the *phytolacca decandra* eating birds he had examined had always been fat.

In answer to inquiries I have the replies of other naturalists stating that poke-berries are fattening to birds, and in no instance have they replied that these berries make birds thin.

The poke-berry (*phytolacca decandra*) is the last of the several important food-berries to ripen in the fall, and is eaten subsequently to the wild grape, sour gum (*nyssa*), dogwood, and the Virginia creeper just before the birds make their southern migration.

It has been suggested to the writer that the period during which the berries are eaten is perhaps too short to affect the birds materially either way, but the evidence, I think, shows otherwise. We are therefore justified in concluding that as an anti-fat to birds *phytolacca* is a failure, and as the physiological function of absorption in man and in birds is practically the same, it is certainly curious that the juice of the berries of the *phytolacca decandra* should have gained such prominence in the treatment of obesity.—*The Hahnemannian Monthly*, December, 1894.

